# Quality of Life and Affective Well-Being in Middle-Aged and Older People with Chronic Medical Illnesses: A Cross-Sectional Population Based Study

**DOI:** 10.1371/journal.pone.0018952

**Published:** 2011-04-29

**Authors:** Anna Wikman, Jane Wardle, Andrew Steptoe

**Affiliations:** 1 Psychobiology Group, Department of Epidemiology and Public Health, University College London, London, United Kingdom; 2 Upper Gastrointestinal Research, Department of Molecular Medicine and Surgery, Karolinska Institutet, Stockholm, Sweden; 3 Health Behaviour Research Centre, Department of Epidemiology and Public Health, University College London, London, United Kingdom; Yale University School of Medicine, United States of America

## Abstract

**Background:**

There has been considerable research into the impact of chronic illness on health-related quality of life. However, few studies have assessed the impact of different chronic conditions on general quality of life (QOL). The objective of this paper was to compare general (rather than health-related) QOL and affective well-being in middle aged and older people across eight chronic illnesses.

**Methods and Findings:**

This population-based, cross-sectional study involved 11,523 individuals aged 50 years and older, taking part in wave 1 of the English Longitudinal Study of Ageing. General QOL was assessed using the CASP-19, happiness was evaluated using two items drawn from the GHQ-12, and depression was measured with the CES-D. Analysis of covariance and logistic regression, adjusting for age, gender and wealth, were performed. General QOL was most impaired in people with stroke (mean 37.56, CI 36.73–38.39), and least in those reporting cancer (mean 41.78, CI 41.12–42.44, respectively), compared with no illness (mean 44.15, CI 43.92–44.39). Stroke (mean 3.65, CI 3.58–3.73) was also associated with the greatest reduction in positive well-being whereas diabetes (mean 3.81, CI 3.76–3.86) and cancer were least affected (3.85, CI 3.79–3.91), compared with no illness (mean 3.97, CI 3.95–4.00). Depression was significantly elevated in all conditions, but was most common in chronic lung disease (OR 3.04, CI 2.56–3.61), with more modest elevations in those with osteoarthritis (OR 2.08, CI 1.84–2.34) or cancer (OR 2.07, CI 1.69–2.54). Multiple co-morbidities were associated with greater decrements in QOL and affective well-being.

**Conclusion:**

The presence of chronic illness is associated with impairments in broader aspects of QOL and affective well-being, but different conditions vary in their impact. Further longitudinal work is needed to establish the temporal links between chronic illness and impairments in QOL and affective well-being.

## Introduction

Quality of life (QOL) for people with chronic physical illnesses such as cancer, diabetes and coronary heart disease (CHD), has been the focus of a substantial body of work. However, the primary focus of research has been on health-related quality of life (HRQOL) rather than positive well-being or QOL in general. The aim of the present study was to investigate the impact of chronic illness using a wider set of QOL constructs, including measures of positive and negative affect, in a general population sample of middle aged and older adults. The main purpose was to establish which conditions have the most pronounced effect, and to discover whether general QOL and affective well-being is especially impaired in different physical illnesses.

HRQOL, however, is just one element of QOL, focusing particularly on the functional effects of the illness and its management on physical, mental and social function. However, poor health does not necessarily equate poor QOL, since some individuals are able to overcome disease-specific limitations, and adapt their lives to pursue their goals. Previous multiple-condition studies of HRQOL have shown that although chronic illness typically has significant negative effects on physical aspects of health, mental health status may remain relatively unaffected [1]–[3]. Less is known about broader aspects of QOL, including issues such as material and emotional well-being, autonomy, self-realisation, control over important aspects of life, and meaning and fulfilment [4], [5]. Positive affect is another important outcome, which has determinants and consequences that are distinct from negative affective states [6], [7]. This general concept of QOL has seldom been assessed in relation to chronic physical illness.

Broader QOL is the focus in the present study, using a measure developed for older adults from a human needs perspective, in which QOL is seen as the satisfaction of needs in four domains, namely control, autonomy, self-realization and pleasure [8]. The control domain refers to people's needs and abilities actively to control their environments, while autonomy is defined as the right of the individual to be free from unwanted interference of others. Self-realization and pleasure capture the active and reflexive processes of being human. Additionally, happiness and depressive symptoms were assessed, so as to explore differential associations with positive and negative affective states. The present analyses were largely exploratory in nature; however, we broadly hypothesised that chronic illnesses would be associated with reduced general QOL, reduced happiness and elevated levels of depressed mood. Multiple chronic illnesses are a particular problem, and a recent study [9] found that people with multiple physical co-morbidities had much poorer HRQOL and higher levels of psychological distress. Therefore, we also hypothesised that multiple chronic physical illnesses would be associated with greater impairment in general QOL and affective well-being than single health conditions, and that a cumulative effect related to the number of chronic illnesses reported would be observed.

This study was carried out using data from the English Longitudinal Study of Ageing (ELSA). This is a representative, population sample of non-institutionalised men and women aged 50 years and over living in England, and includes detailed measures of economic and social circumstances as well as health and well-being. Embedding the study within ELSA ensured that findings were not limited to a selected group of patients with chronic illness, and also made it possible to utilise the detailed measures of wealth and other indicators available in the main study. These provide more robust indicators of socioeconomic status (SES) in this age group than traditional indicators such as educational attainment [10].

## Methods

### Participants

Data for these analyses were from wave 1 of ELSA. This representative, population-based sample of non-institutionalised adults was drawn from people who had previously participated in the Health Survey for England (HSE) in 1998, 1999 or 2001 [11]. The core ELSA sample comprises adults aged 50 years or over, and is drawn from the HSE sample by postcode sector (geographic area), stratified by health authority and proportion of households in non-manual socioeconomic groups. Respondents aged over the age of 50 and living in England in 2002 were used for these cross-sectional analyses (n = 11,523). Comparisons with census data suggest that the ELSA sample is representative of the English population aged 50 and over [12]. Details of the ELSA methodology have been published previously [13], but briefly, wave 1 involved a computer assisted personal interview during a home visit, and a self-completion questionnaire to return by post. The measures used in this study derive both from the home interview and the self-completion questionnaire.

### Ethics statement

Ethical approval for ELSA Wave 1 was granted from the Multicentre Research and Ethics Committee and written informed consent was obtained from all participants.

### Measures

#### Demographics

Since QOL scores tend to vary with age, gender and SES [14], these were included as covariates in all the analyses. Age was initially recorded in exact years, but for the purposes of the analyses, individuals were categorised into 5-year age bands with the exception of the oldest group (50–54; 55–59; 60–64; 65–69; 70–74; 75–79; 80+). Accumulated wealth was used as an indicator of SES, since this best represents economic resources and is a robust measure in this age group [10]. This variable was split into quintiles, 1 being the lowest wealth and 5 the highest wealth.

#### Chronic conditions

Presence of chronic illness was assessed during the home interview, and participants responded to the following question, ‘Has a doctor ever told you that you have (or have had) diabetes, chronic lung disease, asthma, cancer (or history of cancer after age 40), osteoarthritis, rheumatoid arthritis, CHD, or stroke?’.

#### Quality of life and affective well-being

QOL was measured using the CASP-19, a self-completion questionnaire originally developed and validated in a representative UK sample of 263 people in ‘early old age’ (65–75 years) from the Boyd-Orr study [8]. This scale incorporates 19 Likert-scored items relating to perceptions of control, autonomy, self-realization and pleasure representing broad aspects of QOL. The first letter of each domain and its 19 items create the acronym CASP-19 that names the measure. Respondents indicate the degree to which each item applies to them on a scale of ‘never’ (scored 0) to ‘often’ (scored 3), to give a total score out of 57, with higher scores corresponding to better QOL. Representative items include ‘I can do the things I want to do’, ‘I look forward to each day’, ‘I enjoy the things that I do’ and ‘I feel satisfied with the way my life has turned out’. The focus on general aspects of QOL provides distinctive information from health-focused instruments, and the same measure has been used in the Health and Retirement Study (HRS), the Health, Alcohol and Psychosocial factors In Eastern Europe (HAPIEE) study, and the Swedish Longitudinal Occupational Survey of Health (SLOSH). The CASP-19 has been developed as a measure of quality of life in older adults, and a number of studies demonstrate its validity [8]. The Cronbach's α for the CASP-19 in this study was 0.88.

Affective well-being was quantified both in terms of happiness and depression. Respondents completed the 12-item General Health Questionnaire (GHQ-12) [15], a widely used and extensively validated measure both in general and clinical populations worldwide [16]. Happiness was measured with two items drawn from this questionnaire, namely ‘Have you recently been able to enjoy your normal day-to-day activities?’ and ‘Have you recently been feeling reasonably happy, all things considered?’. Each item was rated on a four-point scale (much less than usual, less so than usual, about the same as usual, or more so than usual). For the purpose of these analyses these two items were combined and responses were rated on a continuous scale ranging from 0 to 6, with higher scores indicating higher levels of happiness. The Chronbach's α for this measure was 0.92.

Depressive symptoms were assessed using the Centre for Epidemiologic Studies Depression Scale (CES-D) [17]. A shortened 8-item version with binary response options which was developed for the HRS was used, as described by Steffick [18]. Items refer to the degree to which respondents have experienced depressive symptoms over the past week. Scores range from 0 to 8, with higher scores indicating greater depressive symptoms. The present analyses dichotomised CES-D scores above and below 4, a well-established threshold for indicating the presence or absence of depressed mood [18]. In the present sample the CES-D demonstrated good internal reliability (Cronbach's α = 0.80).

### Statistical analyses

Standard descriptive statistics were used to describe sample characteristics, chronic conditions, QOL and affective well-being. In order to examine whether the eight chronic conditions were associated with differences in affective well-being and general QOL, analysis of covariance was conducted on QOL and happiness ratings, and logistic regressions on depressed mood. Results are presented as means and odds ratios (OR) with 95% confidence intervals (CI) or percentages (%), adjusting for age, gender, and wealth. To protect against spurious results arising from multiple testing, Bonferroni estimates led to setting statistical significance at a p-value of <0.005. The associations of multiple chronic illnesses with QOL and well-being were analysed by categorising people into zero, one, two, three and four or more conditions, and carrying out analysis of covariance on CASP-19 and happiness ratings, and logistic regression on depressed mood.

## Results

Table 1 presents the sample characteristics. The average age of the 11,523 participants included in these analyses was 65.14 (SD 10.47) years, and just over half (54.6%) were women. Complete data on CASP-19 were available for 9,407 participants, happiness ratings were obtained from 10,266 participants, and CES-D scores from 11,063 individuals (representing 82%, 89% and 96% of the sample, respectively). There were marked differences in QOL and affect by age, gender and SES (wealth). The oldest age groups (75–79 and 80+years) had significantly lower CASP-19 scores than the younger age groups (p<0.001), indicating poorer QOL, while women had slightly higher scores than men (mean 42.75, SD 8.67 and, mean 42.21, SD 8.71, respectively). A significant linear association was observed for wealth quintiles, with CASP-19 scores falling from 45.65 (SD 8.70) for the highest quintile to 37.85 (SD 9.44) for the lowest quintile (p<0.001). Depression also varied with age, gender and wealth, with the oldest age groups (70 years and older), women, and those in the bottom two wealth quintiles being significantly more likely to report depressed mood (all at p<0.001). The oldest age group (80+) was less happy than all other age groups (p<0.001). There were no gender differences in happiness ratings (p = 0.75), but those in the lower wealth quintiles reported significantly lower happiness ratings than those in the higher wealth quintiles (p<0.001).

**Table 1:** Participant characteristics.

	Total sample	Quality of life (mean, SD)^1^	Happiness (mean, SD)^2^	Depression (% yes)^3^
**Demographics**				
Mean age (SD)	65.14 (10.47)			
Age, %				
50–54	2074 (18.0)	43.10 (8.62)	3.90 (0.84)	452 (22.3)
55–59	2202 (19.1)	42.86 (8.79)	3.90 (0.83)	483 (22.6)
60–64	1696 (14.7)	43.32 (8.58)	3.93 (0.81)	355 (21.8)
65–69	1718 (14.9)	43.15 (8.44)	3.96 (0.67)	366 (22.1)
70–74	1477 (12.8)	42.58 (8.39)	3.90 (0.77)	355 (24.9)
75–79	1094 (9.5)	41.06 (8.68)	3.86 (0.82)	302 (29.0)
80+	1262 (11.0)	38.83 (8.67)	3.74 (0.86)	379 (33.0)
Gender, %				
Male	5231 (45.4)	42.21 (8.71)	3.90 (0.79)	1002 (20.0)
Female	6292 (54.6)	42.75 (8.67)	3.89 (0.81)	1690 (28.0)
SES (Wealth), %				
Lowest - 1	2192 (19.4)	37.85 (9.44)	3.73 (0.98)	825 (39.7)
2	2262 (20.0)	40.77 (9.12)	3.82 (0.91)	639 (29.6)
3	2265 (20.1)	42.76 (8.10)	3.93 (0.74)	498 (22.8)
4	2253 (20.0)	44.02 (7.69)	3.97 (0.67)	378 (17.3)
Highest - 5	2321 (20.6)	45.65 (8.70)	4.00 (0.67)	311 (13.9)
**Chronic conditions**				
No condition, %	5686 (49.3)			
Diabetes, %	853 (7.4)			
Chronic lung disease, %	749 (6.5)			
Asthma, %	1346 (11.7)			
Cancer, %	676 (5.9)			
Osteoarthritis, %	2183 (18.9)			
Rheumatoid arthritis, %	818 (7.1)			
Coronary heart disease, %	1416 (12.3)			
Stroke, %	509 (4.4)			
**Quality of life and affective well-being**				
Mean quality of life (SD)	42.50 (8.69)			
Mean happiness (SD)	3.89 (0.80)			
Depression, %				
Yes	2692 (24.3)			
No	8371 (75.7)			

Mean (Standard deviation [SD]) and N (%).

1 Quality of life assessed using CASP-19. Scores could range from 0 to 57.

2 Happiness assessed using two items drawn from the GHQ-12, namely ‘Have you recently been able to enjoy your normal day-to-day activities?’ and ‘Have you recently been feeling reasonably happy, all things considered?’. Scores could range from 0 to 6.

3 Depression was assessed using the CES-D. Scores could range from 0 to 8. Total scores = >4 indicate presence of depressed mood (yes), scores = <3 indicate absence of depressed mood (no).

SES = Socioeconomic status.

### Incidence of chronic illness

Almost half of respondents reported at least one chronic condition (Table 1), a comparable rate to that observed in other national surveys including this age range [19]. Osteoarthritis was the most common (18.9%) followed by CHD (12.3%), with stroke and cancer being the least common conditions (4.4% and 5.9%, respectively). Of the 11,523 participants included in these analyses, 3839 (33.3%) reported at least one of the nine conditions, 1445 (12.5%) had two conditions, 417 (3.6%) reported three conditions, 113 (1.0%) had four conditions, and 23 (0.2%) reported five or more co-morbid conditions.

### General quality of life and affective well-being

**Figure 1:**
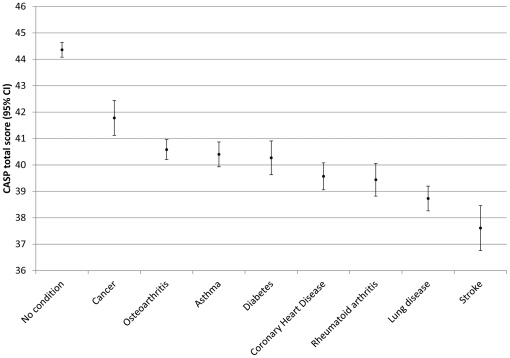
Quality of life scores adjusted for age, gender, and wealth. Mean quality of life scores (95% CI) as measured by the CASP-19, adjusted for age, gender and wealth for respondents with no chronic condition and for all eight chronic conditions. Higher scores indicate better QOL.

There was significantly reduced QOL for each of the eight chronic illnesses (diabetes, lung disease, asthma, cancer, osteoarthritis, rheumatoid arthritis, CHD, stroke), compared with not having any condition (Table 2), adjusting for age, gender, and wealth (all at p<0.005). These findings are illustrated in Figure 1, and indicate that stroke victims had the poorest QOL, followed by chronic lung disease (mean 37.56, CI 36.73–38.39, and mean 38.70, CI 38.07–39.33, respectively). People with rheumatoid arthritis showed similar limitations in QOL as those with CHD, closely followed by diabetes and asthma. QOL was impaired least in people with osteoarthritis and cancer.

**Table 2:** Quality of life and affective well-being for each illness compared with having no chronic illness.

	*Quality of life*		*Happiness ratings*		*Depressed mood*		
*Chronic condition*	n	Mean (95% CI)*	n	Mean (95% CI)*	n	% depressed	OR (95% CI)*
**No condition**	4665	44.15 (43.92–44.39)	4992	3.97 (3.95–4.00)	5364	18.5%	Reference group
**Cancer**	529	41.68 (41.03–42.33)	580	3.85 (3.79–3.91)	630	29.0%	2.07 (1.69–2.54)
**Osteoarthritis**	1750	40.56 (40.18–40.93)	1943	3.75 (3.71–3.78)	2085	31.3%	2.08 (1.84–2.34)
**Asthma**	1072	40.35 (39.88–40.82)	1175	3.80 (3.76–3.84)	1261	33.5%	2.37 (2.06–2.73)
**Diabetes**	620	40.24 (39.63–40.85)	715	3.81 (3.76–3.86)	791	33.1%	2.35 (1.98–2.79)
**Coronary heart disease**	1079	39.59 (39.10–40.08)	1228	3.73 (3.69–3.78)	1334	34.1%	2.44 (2.11–2.83)
**Rheumatoid arthritis**	614	39.43 (38.81–40.04)	697	3.79 (3.73–3.84)	767	38.1%	2.82 (2.39–3.33)
**Chronic lung disease**	585	38.70 (38.07–39.33)	659	3.72 (3.66–3.78)	717	39.7%	3.04 (2.56–3.61)
**Stroke**	345	37.56 (36.73–38.39)	398	3.65 (3.58–3.73)	455	38.4%	2.94 (2.37–3.64)

*Adjusted for age, gender and wealth. All values at p<0.005.

**Figure 2:**
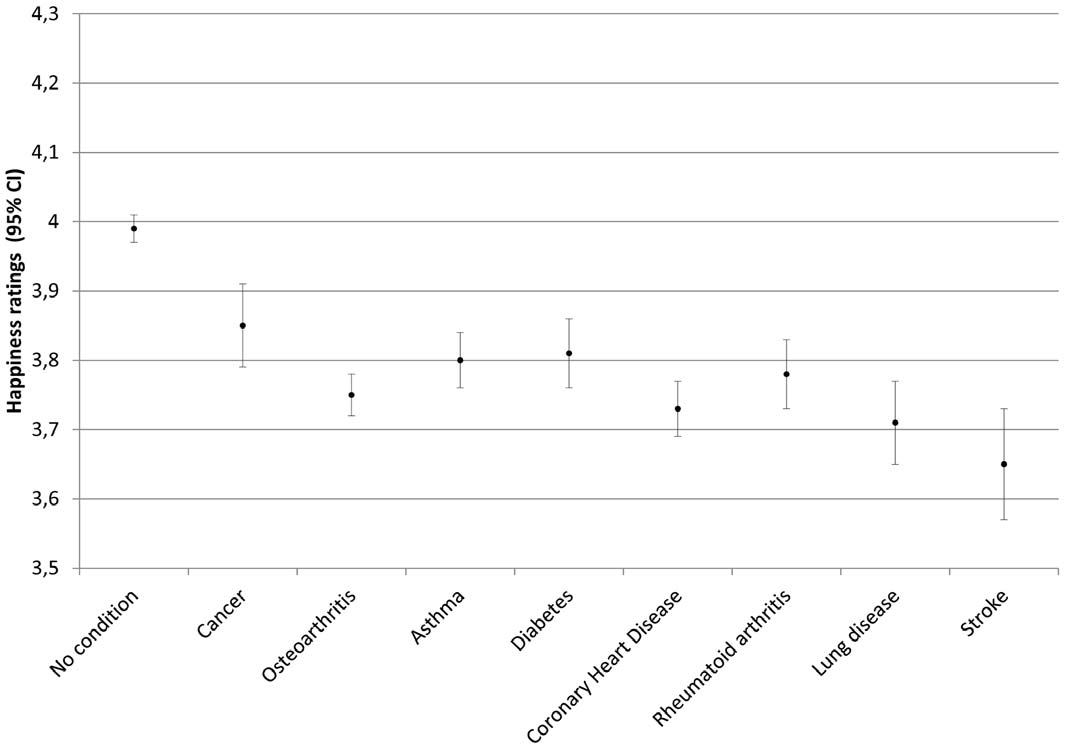
Happiness ratings adjusted for age, gender, and wealth. Mean happiness ratings (95% CI) adjusted for age, gender and wealth for respondents with no chronic condition and for all eight conditions. Higher scores indicate greater happiness.

All eight conditions were associated with significantly reduced happiness ratings (all at p<0.005), controlling for age, gender and wealth (Table 2). People who had had a stroke reported the lowest levels of happiness (mean 3.65, CI 3.58–3.73), followed by those with chronic lung disease, CHD, osteoarthritis and rheumatoid arthritis. People with asthma had similar happiness ratings as those with diabetes. Happiness ratings were least affected in respondents with cancer (mean 3.85, CI 3.79–3.91). These findings are illustrated in Figure 2.

**Figure 3:**
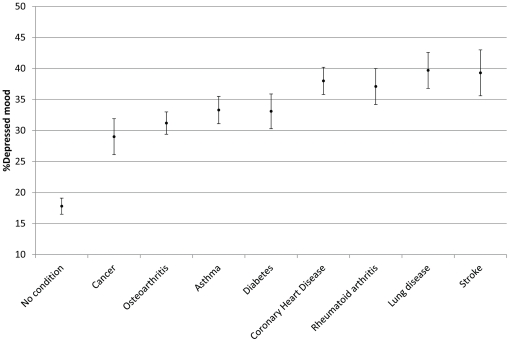
Percentage depressed mood adjusted for age, gender, and wealth. Percentage of depressed mood (95% CI), adjusted for age, gender and wealth, for respondents with no condition and for all eight chronic conditions. Higher scores indicate greater depressed mood.

The proportion of respondents not suffering from any chronic illness who were defined as having depressed mood was 18.5%. The proportion of respondents who were depressed was substantially elevated in all chronic illnesses, controlling for age, gender, and wealth. These findings are illustrated in Figure 3. The highest levels of depression were observed in people with chronic lung disease or stroke who had approximately three times the odds of reporting depressed mood as those without any illness (OR 3.04, CI 2.56–3.61 and, OR 2.94, CI 2.37–3.64, respectively). People with osteoarthritis and cancer had the lowest increased odds for depressed mood (OR 2.08, CI 1.84–2.34 and, OR 2.07, CI 1.69–2.54, respectively) (Table 2).

### Multiple chronic conditions, quality of life and affective well-being

**Figure 4:**
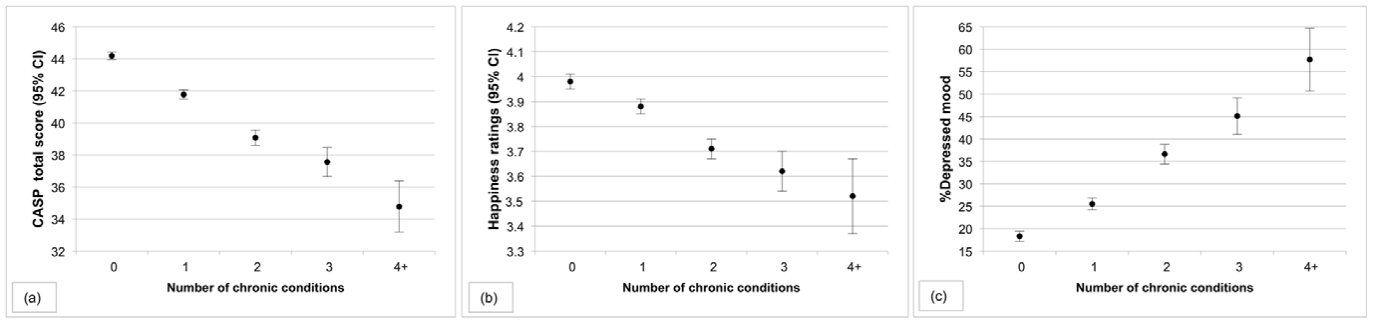
Co-morbidity and quality of life (a), happiness (b) and depressed mood (c). Panel (a) shows the mean quality of life scores (95% CI) for respondents with one, two, three and four or more conditions compared with those without a chronic condition, adjusted for age, gender and wealth. Higher scores indicate better QOL. Panel (b) shows the mean happiness ratings (95% CI) for respondents with no condition compared with one, two, three and four or more co-morbid chronic conditions, adjusted for age, gender and wealth. Higher scores indicate greater happiness. Panel (c) show the percentage of depressed mood, adjusted for age, gender and wealth, for respondents with one, two, three and four or more co-morbid conditions compared with those respondents with no chronic condition.

Adjusting for age, gender and wealth, a significant linear trend was observed across groups in QOL (f _(4, 9216)_ = 148.15, p<0.001, η^2^ = 0.06) with scores decreasing from 44.19 (CI 43.96–44.42) in the no illness group to 34.78 (CI 33.18–36.39) in respondents with four or more co-morbid conditions (Figure 4-a). Similarly, a linear trend for happiness ratings was observed (f _(4, 10059)_ = 44.76, p<0.001, η^2^ = 0.02), with mean values ranging from 3.98 (CI 3.95–4.00) in respondents with no chronic illness to 3.52 (CI 3.37–3.66) in people with four or more conditions (Figure 4-b). However, having three and four or more co-morbid conditions was not associated with significantly worse happiness ratings than were happiness ratings for those with only two co-morbid conditions. Participants with four or more conditions had more than six-fold odds of reporting depressed mood than people with no chronic illness (OR 6.28, CI 4.34–9.08), after controlling for age, gender and wealth. People with three co-morbid chronic illnesses had more than a three-fold increased risk of depressed mood compared with those with no chronic illness (OR 3.66, CI 2.94–4.55). The odds of reporting depressed mood among people with two conditions were 2.66 (CI 2.32–3.04) and the odds of depressed mood in those respondents with one condition only were 1.57 (CI 1.41–1.75) (The percentage of depressed mood by number of chronic conditions is shown in Figure 4-c).

## Discussion

Overall QOL is more than just the presence or absence of disease. Specific functional impairments as measured in HRQOL assessments are likely to contribute to general QOL and affective well-being, but many other factors will be relevant including social and material circumstances, ability to fulfil ambitions, and constraints on opportunities and choice. Assessments of components that constitute general QOL, such as well-being, self-realisation, pleasure, sense of control and autonomy are scarce. In this paper, we studied the association of eight different chronic conditions with general QOL, happiness and depressed mood, in a population-based sample. This study adds to the literature by comparing several conditions while accounting for demographic and social factors. Studying several chronic conditions simultaneously is an essential step towards developing a capacity to assess and rank various chronic diseases for the purpose of prevention and intervention programmes and resource allocation. Assessment of QOL is important in the public health setting. Trends in health expectancies are changing, and contrasting evidence suggest on the one hand that compression of morbidity and a decline in disability in older age has occurred [20], [21], and on the other, an expansion of years of life accompanied by morbidity and function loss [22]. As the population ages, the preservation of QOL in later life is becoming a key policy issue. We found that having a chronic illness was associated with significantly reduced QOL and affective well-being compared with a no disease group, controlling for differences in age, gender and wealth. However, the impact of different illnesses varied, and the ranking of illnesses on the three indicators of QOL and affective well-being was not the same. The findings therefore argue for a more nuanced approach to understanding the impact of chronic illness on the well-being of middle aged and older people.

Previous research has focused largely on HRQOL, using measures such as the Medical Outcomes Study Short Form-36 (SF-36) [23]. Generic HRQOL measures such as this have the advantage of permitting comparisons across chronic illnesses, which is important both for understanding adaptation to different conditions and for resource allocation [24]. A growing body of research has examined HRQOL across a variety of diseases, and many studies have identified significant impairments [25]–[32]. In a population study of over 6000 participants, Hobbs et al [28] compared patients with heart failure, angina, myocardial infarction, hypertension, arthritis and chronic bronchitis. People with heart failure had the poorest HRQOL across most domains, though patients with chronic lung diseases and arthritis also reported poor HRQOL, and people with hypertension had only slight impairments. In the present study, stroke, chronic lung disease (mainly chronic bronchitis and emphysema) and rheumatoid arthritis were associated with the greatest impairments in general QOL and lower positive affect.

A more recent study examined HRQOL in a sample of over 40,000 US army veterans [27]. Presence of diabetes, chronic obstructive pulmonary disease, heart problems, hypertension or arthritis, was associated with significantly lower scores on the physical component summary (PCS) and mental component summary (MCS) of the SF-36. HRQOL differences were more pronounced for the PCS than the MCS in this study, suggesting that although veterans have greater physical and mental HRQOL impairments than the general US population, physical HRQOL is more affected. Chronic lung disease and arthritis were associated with the greatest impairments in HRQOL, whereas scores were only modestly reduced in those with diabetes or hypertension. In our study, diabetes was also associated with somewhat reduced general QOL and happiness ratings, and moderately elevated levels of depressed mood. Other studies have also shown that impact of diabetes on HRQOL is intermediate, relative to other chronic medical conditions [1] and that the effect is more pronounced on the physical relative to the mental health components of the SF-36 [33]. However, these findings are not surprising considering the ‘silent’ nature of illnesses such as diabetes. Using the SF-36, Lyons and colleagues [26] found that, after controlling for age and gender, all patient groups (asthma, diabetes, arthritis, back pain, sciatica, hypertension, angina, myocardial infarction and stroke) had significantly poorer HRQOL than did those without the condition across all the HRQOL domains. In our study, there was also a clear relationship between the number of illnesses and general QOL and well-being, since respondents with multiple chronic conditions had significantly reduced QOL and impaired affective well-being. Previous studies have shown multiple co-morbidities to be associated with feeling unhappy and being more psychologically distressed [9], [34] and that effects may be additive [33].

One explanation for why some chronic illnesses included in the present study have greater impact on QOL and affective well-being than others may be related to the threat to survival. But the impact on well-being was not simply graded by mortality risk, making it likely that some combination of specific impairments related to these conditions and effects on social activities and fulfilment of aspirations also influenced QOL and affective well-being. The significance of these differences in general QOL and affective well-being across conditions for prognosis and illness management may vary. For example, there is strong evidence that depression in patients with CHD is a predictor of recurrent cardiac events and premature mortality [35]. Recent meta-analyses also argue that depression is associated with accelerated mortality in people who have experienced cancer [36], [37]. So even though depression levels were not as high in these conditions as some of the other illnesses tested, modest elevations may nonetheless be important. Less is known about the effects of positive affect on survival in patients with chronic illnesses [6]. More generally, Katon et al [38] have concluded that symptoms of depression and anxiety are associated with greater symptom burden (net of objective disease severity) in a variety of conditions, including chronic lung disease, diabetes, heart disease and arthritis, suggesting that the patterns described here are relevant to adaptation. The relatively modest impact of cancer on psychological distress and general QOL is notable, given the attention that has been focused on these issues in cancer patients [39]. However, it is important to note that the stage of cancer was unknown and that respondents in the present study may have had cancer some years ago, although childhood and young adulthood cancers were excluded. One possible explanation is that benefit finding is prominent among patients with cancer, and may offset in part the negative impact of the condition [40]. Additionally, the types of cancer reported by participants varied widely in this study, and some may have a more adverse impact on QOL and affective well-being than others.

The strengths of this study include the use of a large nationally representative sample of non-institutionalised respondents experiencing a wide range of chronic conditions. Another feature of this study was the use of well-validated measures of general QOL, focusing on broad aspects of QOL rather than on symptomatic and functional impairments, and measures of positive and negative affect to assess affective well-being. Another strength of the study was the high participation rate (>80% for all measures). However, there are some limitations that must be considered. We used the CASP-19 to assess general QOL. Although this measure has been used extensively, the factorial structure remains controversial and alternative versions have been developed [41]. The clinical significance of the differences in general QOL and happiness is not known, since minimal important differences for these measures have not been established. The study was cross-sectional, so causal inferences cannot be drawn. Identification of chronic medical conditions were based on self-report of having been told of the illness by a doctor, which may not be as accurate as physician diagnoses. However, reliability of self-report has been found to be acceptable for conditions that require medical or laboratory diagnostic procedures [42]–[44]. In addition, our analyses did not account for how long these illnesses had been present, or the varying degrees of severity. Duration or onset of illness may have a particular impact on QOL reports, as it seems people continually adapt to their illness. Levels of subjective well-being seem to be especially sensitive to adaptation after the onset of chronic medical conditions. For example, people who might be expected to feel despair given their physical health often report being happier and more satisfied with life than would be expected. Response shift theory [45], [46] postulates that when individuals experience a change in their health status, they may also change their appraisals, internal standards and values regarding QOL. Future work would benefit from considering the influence of response shifts to accurately assess the impact of illness on QOL. Another issue is that the categorisation of chronic illnesses may have disguised important variations. For example, although information was available about different types of cancer, numbers were too small to analyse them separately, so all malignancies were placed in a single category. Additionally, the measure of positive affect was derived from an established index of psychological distress, so was not ideal and likely underestimated the levels of positive affect. Furthermore, selection of illnesses for inclusion in the analyses was based information available in the dataset. Finally, the presence of co-morbidity complicates the question how a specific condition is related to outcome variables such as affect or QOL, and our co-morbidity analyses were performed on a simple count of illnesses rather than on specific disease combinations. Thus, the complex interactions that exist between these chronic conditions have not been accounted for in the present study.

Despite these limitations, the results from this study show that presence of chronic illness is associated with substantially reduced general QOL and happiness, and greater levels of depressed mood across a wide range of conditions suggesting that the presence of chronic illness is not limited to function related impairments only but may have broader implications. Although illnesses associated with significant functional impairment or pain (such as stroke, rheumatoid arthritis and chronic lung disease) were characterized by low mood, low positive affect and poor QOL, it is notable that levels of happiness were not greatly impaired in those with cancer, and only modestly impaired in those with diabetes, asthma or rheumatoid arthritis. These findings suggest that measures of positive affect may reveal subtle effects of physical illness that are not addressed by other measures of well-being and attention should be directed to examination of reduced positive affect as well as negative affective states such as depression. Further longitudinal work is needed to establish the temporal links between chronic illness and impairment in QOL and positive well-being.
